# Spectrally Tunable Lead-Free Perovskite Rb_2_ZrCl_6_:Te for Information Encryption and X-ray Imaging

**DOI:** 10.3390/ma17112530

**Published:** 2024-05-24

**Authors:** Guoxue Pan, Mingqing Li, Xiaotong Yu, Yuanhao Zhou, Minghui Xu, Xinxin Yang, Zhan Xu, Qianli Li, He Feng

**Affiliations:** School of Materials Science and Engineering, Shanghai University, Shanghai 200444, China; panguoxue@shu.edu.cn (G.P.); mingqing114@foxmail.com (M.L.); yxt1998@shu.edu.cn (X.Y.); zhouyuanhao1998@shu.edu.cn (Y.Z.); minghuixyv@shu.edu.cn (M.X.); yangxinxin@t.shu.edu.cn (X.Y.); xuzhan@shu.edu.cn (Z.X.)

**Keywords:** lead-free perovskite, Rb_2_ZrCl_6_:Te, spectrum-tunable, information encryption, X-ray imaging

## Abstract

A series of lead-free Rb_2_ZrCl_6_:*x*Te^4+^ (*x* = 0%, 0.1%, 0.5%, 1.0%, 2.0%, 3.0%, 5.0%, 10.0%) perovskite materials were synthesized through a hydrothermal method in this work. The substitution of Te^4+^ for Zr in Rb_2_ZrCl_6_ was investigated to examine the effect of Te^4+^ doping on the spectral properties of Rb_2_ZrCl_6_ and its potential applications. The incorporation of Te^4+^ induced yellow emission of triplet self-trapped emission (STE). Different luminescence wavelengths were regulated by Te^4+^ concentration and excitation wavelength, and under a low concentration of Te^4+^ doping (*x* ≤ 0.1%), different types of host STE emission and Te^4+^ triplet state emission could be achieved through various excitation energies. These luminescent properties made it suitable for applications in information encryption. When Te^4+^ was doped at high concentrations (*x* ≥ 1%), yellow triplet state emission of Te^4+^ predominated, resulting in intense yellow emission, which stemmed from strong exciton binding energy and intense electron-phonon coupling. In addition, a Rb_2_ZrCl_6_:2%Te^4+^@RTV scintillating film was fabricated and a spatial resolution of 3.7 lp/mm was achieved, demonstrating the potential applications of Rb_2_ZrCl_6_:*x*Te^4+^ in nondestructive detection and bioimaging.

## 1. Introduction

Perovskite had basic and practical significance in photoelectric research, such as solar power, information encryption, and scintillation applications [1]. There were many types of perovskite, which could be divided into Pb-based perovskites [2,3,4,5,6] and non-Pb perovskites [7,8,9,10] according to whether the compound contained lead (Pb). Lead halide perovskites (LHPs) garnered significant research interest in the optoelectronic field, including solar cells, LEDs, and anti-counterfeiting [11,12,13,14,15], due to their excellent light absorption properties, tunable bandgap width, and high photoluminescence quantum yield (PLQY). LHP nanocrystals were identified as high-performance scintillators due to their cost-effectiveness, tunable emission, and fast decay time [16,17]. However, unfortunately, the large-scale commercial application of this material was hindered by stability and toxicity issues [18]. Therefore, lead-free perovskite materials that displayed non-toxicity, stability, and exceptional luminescent properties became the focus of significant attention, emerging as a primary area of research. In recent years, researchers developed numerous lead-free perovskite materials by substituting lead with various low-toxicity and non-toxic elements, for example, tin-based [19,20], copper-based [21,22,23,24,25], manganese-based [26], and zirconium-based perovskites [27,28].

Due to its stability and distinctive luminescent properties, the all-inorganic Rb_2_ZrCl_6_ attracted widespread attention. Zhang synthesized Rb_2_ZrCl_6_ microcrystals via a hydrothermal method and utilized them as phosphors to prepare WLEDs [29]. The Rb_2_ZrCl_6_ microcrystals exhibited blue-white emission characteristics, with a high PLQY value of up to 60%. Notably, they demonstrated significant stability when exposed to heat, ultraviolet light, and environmental conditions involving water/oxygen. Zheng pioneered the environmentally friendly wet grinding method to synthesize a series of highly crystalline Rb_2_ZrCl_6_:*x*Te^4+^ microcrystals. Furthermore, the research indicated that the effective emission of these perovskite materials could be attributed to their direct bandgap characteristics and zero-dimensional structure [30]. Shi introduced the tunable emission characteristics of Rb_2_ZrCl_6_: *x*Sb^3+^ phosphors that depend on the different excitation wavelengths [31]. In 2023, Liu synthesized Te^4+^ doped Rb_2_ZrCl_6_ microcrystals, and conducted a preliminary study on their optical properties to manufacture white light emitting diodes [32]. These studies on Rb_2_ZrCl_6_ proved that it has excellent luminescence properties.

However, the emission of Rb_2_ZrCl_6_ required excitation at high-energy wavelengths, posing a challenge for effective photon excitation when used in conjunction with commercial UV chips and electroluminescent devices [33]. Furthermore, the single emission of Rb_2_ZrCl_6_ could not meet the requirements of multiple anti-counterfeiting and information encryption applications. Therefore, the development of an effective wavelength tuning technique for Rb_2_ZrCl_6_ was of significant importance in expanding its applications. Doping technology emerged as an effective means of tuning the luminescent properties of perovskite halides. Intelligent light-emitting materials achieved an intelligent response through photochromism [34]. Photochromism entailed emitting different colors of light under various excitation wavelengths. Materials with photochromic properties were excellent candidates for anti-counterfeiting applications where light of different colors was emitted under different excitation wavelengths. It has been reported that Rb_2_ZrCl_6_:Te^4+^ exhibited characteristics of emitting blue and yellow light at 255 nm and 388 nm, respectively, making it a promising material for anti-counterfeiting applications [32]. In addition, Rb_2_ZrCl_6_ was a scintillator material with potential. However, the anti-counterfeiting of photochromism applications and scintillation properties of Rb_2_ZrCl_6_ powder samples had not been studied systematically.

In this study, the anti-counterfeiting application of Rb_2_ZrCl_6_:Te^4+^ was explored and the luminescence mechanism of Rb_2_ZrCl_6_:Te^4+^ was studied in detail, and its application in anti-counterfeiting and biological imaging was expanded and innovated. Rb_2_ZrCl_6_:*x*Te^4+^ microcrystals were synthesized through a hydrothermal method, and the crystal structure and spectral characteristics of Rb_2_ZrCl_6_:*x*Te^4+^ were analyzed. Remarkably, this modification resulted in the emergence of an additional yellow emission when subjected to low-energy excitation at 388 nm, facilitating the transition from the initial blue emission to a vibrant yellow emission. We designed an anti-counterfeiting information encryption and decryption experiment using Rb_2_ZrCl_6_:0.1%Te^4+^ powder to emit different colors under different excitations. Simultaneously, X-ray imaging tests were conducted on scintillating films of Rb_2_ZrCl_6_@RTV and Rb_2_ZrCl_6_:2%Te^4+^@RTV, and both demonstrated imaging capabilities. This discovery demonstrated the potential applications of Rb_2_ZrCl_6_:*x*Te^4+^ in information encryption, anti-counterfeiting, and bioimaging.

## 2. Experiment Section

### 2.1. Materials

Rubidium chloride (RbCl, 99.95%, CN), zirconium chloride (ZrCl_4_, 99.9%, CN), and antimony chloride (TeCl_4_, 99%, CN) were purchased from Aladdin Ltd. Hydrochloric acid (CP, 36.0~38.0%, CN) and methanol (GR, 99.7%, CN) was purchased from Sinopharm Chemical Reagent Co, Ltd. None of the materials required further purification.

### 2.2. Synthesis of Rb_2_ZrCl_6_:xTe^4+^ Microcrystals

**Figure 1:**
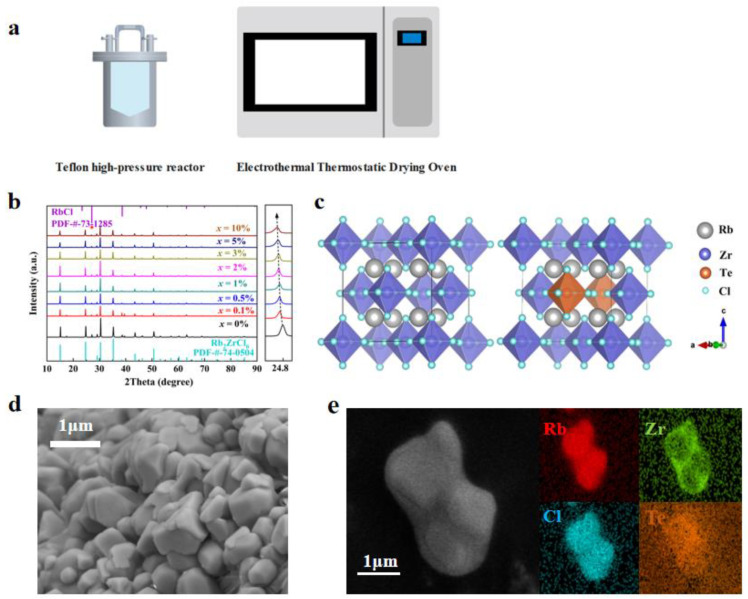
(**a**) Schematic diagram of the synthesis method. (**b**) XRD patterns of Rb_2_ZrCl_6_:*x*Te^4+^ (*x* = 0%, 0.1%, 0.5%, 1.0%, 2.0%, 3.0%, 5.0%, 10.0%) [35]. (**c**) Crystal structure diagram of Rb_2_ZrCl_6_ and Rb_2_ZrCl_6_:2%Te^4+^. (**d**) SEM image, and (**e**) the elemental mappings of Rb_2_ZrCl_6_:2%Te^4+^ microcrystals.

The synthesis of Rb_2_ZrCl_6_:*x*Te^4+^ involved a conventional hydrothermal method. Figure 1a schematically illustrates the Rb_2_ZrCl_6_:*x*Te^4+^ growth method. Specifically, 2 mmol of RbCl, 1 − *x* mmol of ZrCl_4_ and *x* mmol TeCl_4_ were dissolved in 5 mL of HCl and placed in a 25 mL Teflon high-pressure reactor. After being heated at 180 °C for 12 h, the solution was gradually cooled for over 8 h to reach room temperature. Rb_2_ZrCl_6_:*x*Te^4+^ microcrystals were observed to form once the temperature reduced to room temperature. Subsequently, Rb_2_ZrCl_6_:*x*Te^4+^ microcrystals were subjected to three methanol washes and then dried in an oven at 80 °C for a total for 6 h.

### 2.3. Preparation of Rb_2_ZrCl_6_@RTV and Rb_2_ZrCl_6_:2%Te^4+^@RTV Scintillation Screens

The Rb_2_ZrCl_6_ powder was thoroughly mixed with RTV in a 2:1 ratio, and the Rb_2_ZrCl_6_@RTV scintillation film was fabricated using the screen-printing method. The production process of the Rb_2_ZrCl_6_:2%Te^4+^@RTV rigid scintillation screen follows the same method as that of the Rb_2_ZrCl_6_@RTV scintillation film.

### 2.4. Measurement and Characterization

The X-ray diffraction (XRD) diffractometer utilized in this study was the PANalytical instrument (Almelo, The Netherlands) employed for polycrystalline powder characterization. The testing conditions included a tube voltage set at 40 kV, with a Cu target material selected, emitting radiation at a wavelength of 1.540598 Å. Scanning was conducted within an angular range of 10° to 90° at a rate of 6°/min, with a step size of 0.02°. Scanning Electron Microscope (SEM) image measurements were performed using an SU3500 from Hitachi, Tokyo, Japan, and the samples were subjected to energy dispersive spectrometer (EDS) elemental mapping using a scanning electron microscope (Zeiss Gemini SEM 300, ZEISS, Jena, Germany). In this study, the experimental apparatus set to a voltage of 5 kV. PL, time-resolved photoluminescence (TRPL), and PLQY measurements were conducted using a PL spectrometer (Edinburgh, FLS 1000, Livingston, UK). Thermo-gravimetric (TG) data and Differential Scanning Calorimetry (DSC) data were collected with the aid of a Netzsch instrument (STA 449 F5 Jupiter, Burlington, MA, USA). The temperature range spanned from room temperature to 1273 K, with a heating rate of 10 K/min, under an Ar_2_ atmosphere for testing. The UV–VIS spectrum was obtained using a UV–VIS spectrophotometer (HITACHI, U-3900H). The imaging system used in this study was a custom-built platform consisting of an X-ray source, a scintillator screen, a CCD detector, as well as image processing and display equipment. The experimental setup involved a voltage of 100 kV, a current of 1000 μA, and a peak energy of 165 kVp.

## 3. Results and Discussion

Rb_2_ZrCl_6_:*x*Te^4+^ microcrystals were synthesized via a hydrothermal method, and the structural properties of Rb_2_ZrCl_6_:*x*Te^4+^ were characterized. Phase analysis of Rb_2_ZrCl_6_:*x*Te^4+^ was conducted, followed by an examination of the structural changes in the crystal before and after doping. Figure 1 presents the test results of structural performance characterization.

Figure 1b demonstrates the XRD patterns of Rb_2_ZrCl_6_:*x*Te^4+^ samples. Both the hydrothermally synthesized Rb_2_ZrCl_6_ and Rb_2_ZrCl_6_:*x*Te^4+^ samples exhibited a common hybrid peak at a diffraction angle of 27.072° [36]. When compared with the PDF card, this hybrid peak precisely matches the diffraction peak of RbCl (200) [31], which is not luminous. Due to the ionic radius of Te^4+^ being larger than Zr^4+^, the substitution of Te^4+^ for Zr^4+^ results in lattice expansion. Consequently, the diffraction peak position at (220) shifts notably towards smaller angles with increasing Te^4+^ content. In order to ascertain the successful incorporation of Zr into the lattice of Rb_2_ZrCl_6_, calculations of the lattice constants of the material were performed using the WinCSD software (Version 4). The computed results indicated that with the introduction of Zr, the lattice parameter of the material increased from 7.14081 Å at *x* = 0 to 7.17377 Å at *x* = 10%. As illustrated in Figure 1c, the Rb_2_ZrCl_6_ perovskite crystal structure is a typical cubic phase. Unlike the conventional 3D perovskite ABX_3_, Rb_2_ZrCl_6_ features a vacancy-ordered structure composed of [ZrCl_6_]^2−^ octahedra and Rb^+^ ions. Te^4+^ replaces the Zr^4+^ sites. The Zr^4+^ in Rb_2_ZrCl_6_ substitutes for B^2+^ sites, leading to a 50% periodic vacancy in [ZrCl_6_]^2−^. This causes the octahedral clusters to be fully separated by the A-site cation Rb^+^, resulting in a distinct 0D perovskite structure. To gain a comprehensive understanding of the sample’s morphology and elemental distribution, perovskite microcrystalline powder was meticulously examined through SEM and EDS. As depicted in Figure 1d, the SEM image reveals that the perovskite exhibits a microcrystalline tetrahedral structure, and the average size of Rb_2_ZrCl_6_:2%Te^4+^ microcrystals was 2 μm. Moreover, in Figure 1e, the mapping of Cs, Zr, and Sb elements in a single crystal revealed that the Te^4+^ dopants were homogeneously introduced into the Rb_2_ZrCl_6_ host lattice.

**Figure 2:**
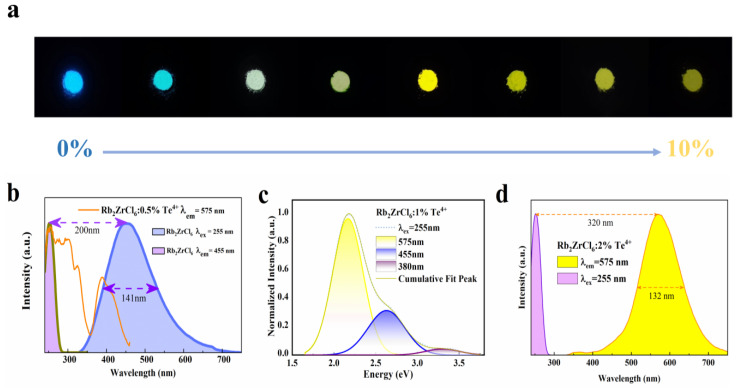
Optical characterizations of Rb_2_ZrCl_6_:*x*Te^4+^. (**a**) Photographs of the Rb_2_ZrCl_6_:*x*Te^4+^ powders under 254 nm UV light. (**b**) PL and PLE spectra of Rb_2_ZrCl_6_. (**c**) Schematic diagram of 255 nm excitation spectra Gaussian fitting process of Rb_2_ZrCl_6_:1%Te^4+^. (**d**) PL and PLE spectra of Rb_2_ZrCl_6_:2%Te^4+^.

Next, the optical properties of Rb_2_ZrCl_6_:*x*Te^4+^ were investigated. The ultraviolet fluorescence spectrum of Rb_2_ZrCl_6_:*x*Te^4+^ showed diverse luminescent characteristics under various excitation wavelengths—bright blue at 254 nm excitation and bright yellow at 365 nm excitation, which resulted in a luminescence wavelength range that could be adjusted within 455 nm to 575 nm, transitioning from blue to yellow, demonstrating excellent spectral tunability. Figure 2 displays the results of optical property testing.

As shown in Figure 2a, the precise control of the Te^4+^ doping level allowed for effective spectral tuning can result in the transformation from blue to yellow light emission.

As shown in Figure 2b, the emission spectrum of Rb_2_ZrCl_6_ at 255 nm partially overlaps with that excitation spectrum of Rb_2_ZrCl_6_:0.5%Te^4+^ at 575 nm, indicating that the blue emission of Rb_2_ZrCl_6_ can be absorbed by Te^4+^. The distinctive emission peak of Rb_2_ZrCl_6_ is situated at 455 nm, featuring a considerably wide full-width at half-maximum (FWHM) of 141 nm. Calculations reveal a substantial Stokes shift of 200 nm. The presence of such a substantial Stokes shift, along with the broad spectral characteristics, strongly implies that the PL mechanism in Rb_2_ZrCl_6_ may indeed originate from the emission of self-trapped excitons [37]. As shown in Figure S1, The UV–VIS absorption spectrum of Rb_2_ZrCl_6_:*x*Te^4+^ reveals a broad absorption range spanning from 250 to 475 nm. Compared to Rb_2_ZrCl_6_, the Te^4+^ doped Rb_2_ZrCl_6_ sample exhibits significantly enhanced absorption features in the wavelength range of 370–425 nm.

Figure 2c depicts the Gaussian peak-splitting curve for Rb_2_ZrCl_6_:1%Te^4+^. Under 255 nm excitation, three distinct peaks are readily apparent. Notably, the peak at 575 nm exhibits the highest intensity, corresponding to the ^3^P_1_→^1^S_0_ triplet energy transition of Te^4+^. The peaks at 380 nm and 455 nm originate from the ^1^P_1_→^1^S_0_ singlet transition of Te^4+^ and the STE emission of Rb_2_ZrCl_6_, respectively [32].

The characteristic emission peak of Rb_2_ZrCl_6_:2%Te^4+^ is centered at 575 nm, exhibiting a noteworthy full width at a half-peak of 132 nm, and a calculated Stokes shift of 187 nm (Figure 2d).

**Figure 3:**
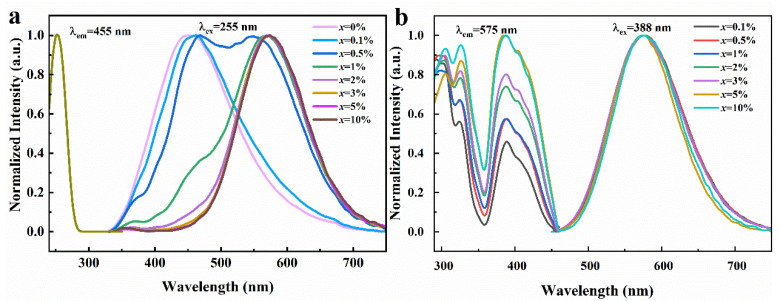
(**a**) Normalized PLE and PL spectra of Rb_2_ZrCl_6_:*x*Te^4+^ (λ_em_ = 455 nm and λ_ex_ = 255 nm). (**b**) Normalized PLE and PL spectra of Rb_2_ZrCl_6_:*x*Te^4+^ (λ_em_ = 575 nm and λ_ex_ = 388 nm).

As depicted in Figure 3a, when excited under 255 nm UV light, the emission spectrum curves of Rb_2_ZrCl_6_ exhibit distinct variations based on different Te^4+^ doping concentrations. Rb_2_ZrCl_6_ itself exhibits an emission peak at 455 nm, while the introduction of Te^4+^ results in the emergence of a novel emission peak at 575 nm. As shown in Figure S2, as the concentration of Te^4+^ doping is increased, the intensity of the 455 nm peak gradually diminishes until it eventually fades away, while the 575 nm peak continues to rise until it reaches a maximum, after which it gradually decreases with higher doping ion concentrations. Therefore, the optical performance test in this paper is mainly based on Rb_2_ZrCl_6_:2%Te^4+^. It can be seen from Figure 3a that the two emission peaks change regularly with different doping concentrations, and the energy transfer between Rb_2_ZrCl_6_ and Te^4+^ is observed.

As depicted in Figure 3b, under UV excitation of 388 nm, Rb_2_ZrCl_6_ with different Te^4+^ doping concentrations presents a consistent emission peak at 575 nm. When the excitation and emission spectra of Rb_2_ZrCl_6_:*x*Te^4+^ are characterized, it is found that the excitation peak of 388 nm is detected at the emission wavelength of 575 nm, and this excitation peak is from the triplet emission of Te^4+^ [32].

**Figure 4:**
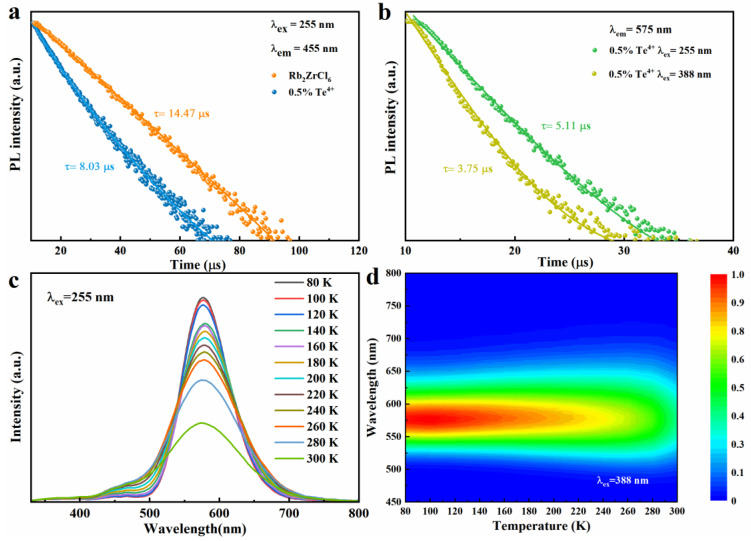
(**a**) PL decay curve of Rb_2_ZrCl_6_ and Rb_2_ZrCl_6_:0.5%Te^4+^, respectively. (**b**) PL decay curve of Rb_2_ZrCl_6_:0.5%Te^4+^. (**c**) Temperature-dependent PL spectra of Rb_2_ZrCl_6_:1%Te^4+^ (λ_ex_ = 255 nm). (**d**) Pseudo map of Rb_2_ZrCl_6_:1%Te^4+^ PL spectra at low temperature (T = 80–300 K, λ_ex_ = 388 nm).

The decay times of Rb_2_ZrCl_6_ and Rb_2_ZrCl_6_:0.5%Te^4+^ PL at room temperature are studied, as shown in Figure 4a. The lifetime of Rb_2_ZrCl_6_ is about 14.47 μs (λ_ex_ = 255 nm, λ_em_ = 455 nm), and the lifetime of Rb_2_ZrCl_6_:0.5%Te^4+^ is reduced to 8.03 μs (λ_ex_ = 255 nm, λ_em_ = 455 nm) compared to Rb_2_ZrCl_6_. This is due to the partial transfer of the exciton of Rb_2_ZrCl_6_ to the Te^4+^ level, which would have led to the reduction of the luminous exciton of Rb_2_ZrCl_6_, resulting in faster decay. This also corresponds to the results of excitation and emission spectra as shown in Figure 3a. As shown in Figure 4b, the lifetimes of Rb_2_ZrCl_6_:0.5%Te^4+^ (λ_ex_ = 255 nm, λ_em_ = 575 nm) and Rb_2_ZrCl_6_:0.5%Te^4+^ (λ_ex_ = 388 nm, λ_em_ = 575 nm) are 5.11 μs and 3.75 μs, respectively. This is because under the 388 nm excitation, luminous excitons act directly on Te^4+^. Under 255 nm excitation, the excitons acting on Te^4+^ are partly derived from Rb_2_ZrCl_6_, so the lifetime is longer.

Under 255 nm ultraviolet excitation, Rb_2_ZrCl_6_:1%Te^4+^ exhibits peaks at 455 nm and 575 nm, testing the temperature-dependent (low-temperature) PL curve of Rb_2_ZrCl_6_:1%Te^4+^, as illustrated in Figure 4c. The PL spectra obtained under 255 nm excitation exhibit a trend where the PL intensity of the 1%Te^4+^ doped Rb_2_ZrCl_6_ increases as the temperature decreases. A similar change in trend was observed for samples excited at 388 nm (Figure S3). This behavior can be attributed to the reduction in electron–phonon coupling at lower temperatures, which is more conducive to the inter-system crossing (ISC) process, consequently leading to an enhancement in the strength of the triplet STE [38]. Figure 4d presents a false-color representation of the changes in PL intensity and wavelength in response to temperature variations for Rb_2_ZrCl_6_:1%Te^4+^ when excited with a 388nm light source. For a similar investigation under 255 nm excitation, please refer to Figure S4, which illustrates the pseudo-color diagram depicting PL intensity and wavelength changes as a function of temperature for Rb_2_ZrCl_6_:1%Te^4+^. As the temperature rises, there is a gradual reduction in the PL intensity attributed to heat-induced non-radiative recombination. Simultaneously, an increase in temperature is associated with a widening of the FWHM of the PL spectrum. This phenomenon indicates that at elevated temperatures, greater exciton–phonon coupling is at play, thereby promoting non-radiative recombination processes [39].

Next, in this study, the temperature-dependent relationship of FWHM and PL integral strength was analyzed. Band structure calculations and DOS were performed on Rb_2_ZrCl_6_:Te, Rb_2_ZrCl_6_ had a large band gap of 3.69 eV. After doping Te^4+^, a new impurity level was introduced, and the band gap was 3.47 eV. We analyzed the luminescence mechanism of Rb_2_ZrCl_6_:Te, in which yellow ^1^S_0_→^3^P_1_ emission originated from the triplet emission of Te^4+^. Figure 3 displays the results.

**Figure 5:**
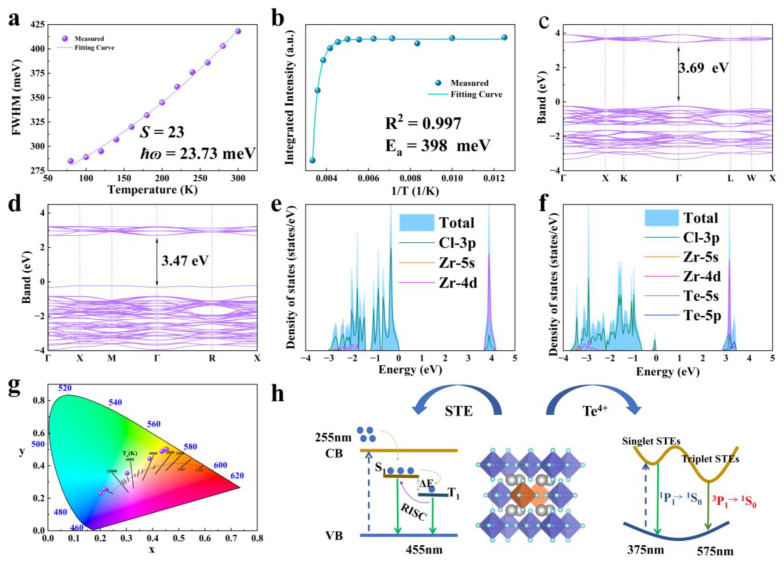
(**a**) Temperature dependence curve of FWHM for the 575 nm luminescence peak of Rb_2_ZrCl_6_:2%Te^4+^. (**b**) Temperature-dependent curve of integral strength of PL curve. (**c**,**d**) Band structures of Rb_2_ZrCl_6_ and Rb_2_ZrCl_6_:2%Te^4+^, respectively. (**e**,**f**) DOS of Rb_2_ZrCl_6_ and Rb_2_ZrCl_6_:2%Te^4+^, respectively. (**g**) CIE color coordinates of the eight Rb_2_ZrCl_6_:*x*Te^4+^ samples under 255 nm excitation. (**h**) Schematic diagram of energy transfer process of Rb_2_ZrCl_6_:Te.

As depicted in Figure 5a, as the optimal doping concentration was determined to be 2% Te^4+^, the temperature-dependent curve of FWHM of the emission peak at 575 nm for Rb_2_ZrCl_6_:2%Te^4+^ was fitted to the phonon broadening model. The equation is provided below:

(1) $$FWHM\left(T\right)=2.36\sqrt{S}\hbar \omega_{phonon}\sqrt{coth\left(\frac{\hbar \omega_{phonon}}{2k_{B}T}\right)}$$

where *S* is the electron–phonon coupling, the *ħɷ* phonon is the phonon frequency, and *k_B_* is the Boltzmann constant. The fitting results are shown in Figure 5a. The Huang–Rhys factor (*S*) is found to be 23, the *ħɷ* phonon is calculated to be 23.73 meV, and this relatively high *S* value indicates the presence of a robust electron–phonon coupling in Rb_2_ZrCl_6_:*x*Te^4+^. Figure 5b shows the temperature-dependent curve of integral strength of the PL curve. It is shown that the luminescent intensity of Rb_2_ZrCl_6_:2%Te^4+^ microcrystals demonstrates a decrease as the temperature rises, indicating the occurrence of thermal quenching of luminescence (Figure 5b). The temperature dependency of PL intensity can be aptly described using the Arrhenius formula fitting [40]:

(2) $$I\left(T\right)=\frac{I_{0}}{1+Ae^{\frac{-E_{a}}{k_{B}T}}}$$

In the Arrhenius formula, *I*_0_ represents the PL intensity at 0 K, *E_a_* stands for the activation energy, and *k_B_* denotes the Boltzmann constant. The *E_a_*, determined through Arrhenius formula fitting, is calculated to be 398 meV. Notably, this *E_a_* value significantly exceeds the thermal energy at room temperature, suggesting the formation of a stable STE with highly localized characteristics [41].

In Figure 5c, the Rb_2_ZrCl_6_ band results show the calculation results. The exchange-correlation functional employed the generalized gradient approximation (GGA), with a cutoff energy set at 550 eV. Brillouin zone sampling was achieved using a 4 × 4 × 4 Monkhorst-Pack κ-point grid. The convergence criteria for structural optimization were met when the energy per atom was below 10^−8^ eV and the forces were less than 10^−3^ eV/Å, which shows that Rb_2_ZrCl_6_ has a direct band gap of 3.69 eV, after doping 2%Te^4+^, it becomes 3.47 eV, as shown in Figure 5d. According to Figure 5e, the CBM is mainly composed of Zr 4d orbitals, supplemented by a small contraction of the Cl 3p orbital, while the VBM is mainly Cl 3p orbitals. As shown in Figure 5f, after doping with Te^4+^, an impurity level is introduced with the top 3.47 eV of the valence band, including the Te 5s and Cl 3p orbitals. The CBM is occupied by the Zr 4d, Cl 3p, and Te 5p orbitals.

By DOS analysis, Rb does not play a role in bandgap formation. This implies that electron bands predominantly arise from zirconium and halogen contributions within isolated [ZrCl_6_]^2−^octahedrons. The movement of electrons and holes is confined in three spatial dimensions, leading to the emergence of a 0D electronic structure. This unique structure fosters efficient emission of STEs [30]. Figure 5g depicts the CIE color coordinates of Rb_2_ZrCl_6_:*x*Te^4+^. Notably, these coordinates exhibit a systematic shift, transitioning from the cold white light region (0.20, 0.23) to the warm white light region (0.45, 0.50) as the Te^4+^ concentration increases. This shift in color coordinates demonstrates the tunable nature of the emission spectrum.

Based on the discussion above, the mechanism of STEs for Rb_2_ZrCl_6_:Te under UV excitation was obtained, as shown in Figure 5h. When stimulated by light, Rb_2_ZrCl_6_:Te^4+^ will undergo lattice distortion, resulting in STE emission. Owing to the narrow bandgap between the S_1_ and T_1_ states generated by STEs, the triplet exciton undergoes thermal activation to the vibrational level of the singlet state through RISC. Subsequently, it undergoes radiative transition from the S_1_ state to the ground state, emitting photons and resulting in a blue emission at 455 nm under 255 nm excitation. The introduction of Te^4+^ as a dopant creates new energy levels. Additionally, the formation of [TeCl_6_]^2−^ octahedra induces Jahn–Teller distortion, further enhancing the generation of self-trapped excitons. Electrons are excited from the valence band of Rb_2_ZrCl_6_ to the conduction band, followed by a nonradiative relaxation process. This relaxation results in a dual outcome: one portion leads to bulk STE emission, specifically in the form of blue light emission, while the other fraction transitions to the Te^4+^ energy levels. In the case of Te^4+^, its ground state is categorized as the ^1^S_0_ energy level, and its excited state comprises three triplet energy levels, namely ^3^P_0_, ^3^P_1_, and ^3^P_2_, in addition to a singlet energy level denoted as ^1^P_1_. Based on transition rules, transitions from ^1^S_0_ to ^1^P_1_ and ^3^P_1_ are permitted. Transitions from ^1^S_0_ to ^3^P_0_ and ^1^S_0_ to ^3^P_2_ are entirely prohibited at the electric dipole transition level. Early studies indicate that the low-energy excitation region of Te^4+^ originates from the ^1^S_0_→^3^P_1_ transition [42], with the absorption peaks in the 370–425 nm range attributed to the ^1^S_0_→^3^P_1_ transition. Under 388 nm ultraviolet excitation, the 575 nm emission of Rb_2_ZrCl_6_:Te arises from the triplet transition of ^1^S_0_→^3^P_1_. Under 255 nm, some electrons in the singlet state ^1^P_1_ transition back to the ground state, resulting in PL at approximately 380 nm. Meanwhile, the remaining electrons undergo a process known as ISC, transitioning from the singlet state ^1^P_1_ to the triplet state ^3^P_1_. Subsequently, under 388 nm, the triplet state relaxes back to the ground state, emitting a yellow light at 575 nm. The PLQY for Rb_2_ZrCl_6_ at room temperature is notably high, reaching 62.64%. Figure S5 illustrates that the PLQY remains at 44.74% at room temperature even with a 2% Te^4+^ dopant.

**Figure 6:**
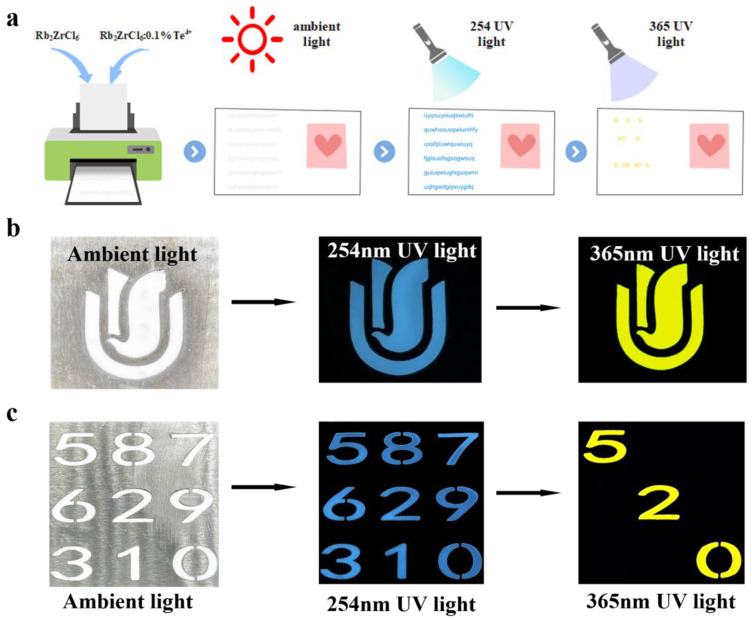
(**a**) A Schematic representation of the concept of information encryption and decryption. (**b**) Anti-counterfeiting of Rb_2_ZrCl_6_:0.1%Te^4+^ pattern. (**c**) Information encryption and decryption test.

Next, Rb_2_ZrCl_6_:2%Te^4+^ was tested for stability, and exhibited good air stability over a period of four months. Moreover, the dual-state emission characteristic provides theoretical support for research and experimental design in information encryption applications. Figure 6 showed the wide application prospect of Rb_2_ZrCl_6_:2%Te^4+^ by designing anti-counterfeiting and information encryption experiments.

Using Rb_2_ZrCl_6_:0.1%Te^4+^ spectral adjustable properties, different colors were displayed through different wavelengths to achieve the function of anti-counterfeiting and information encryption. Figure 6a is a schematic diagram of the concept of information encryption and decryption. Figure 6b shows an example of anti-counterfeiting. The Rb_2_ZrCl_6_:0.1%Te^4+^ powder pattern appeared white under ambient light; at 254 nm UV light, it emitted blue light, and at 365 nm UV light, it emitted yellow light, which could be clearly distinguished by the naked eye. In order to further enhance the security level of optical information encryption, an optimization scheme was designed based on the dual excitation source of Rb_2_ZrCl_6_:0.1%Te^4+^ and the emission band luminescent material. As shown in Figure 6c, the “520” numbers were Rb_2_ZrCl_6_:0.1%Te^4+^ powder, and the rest were Rb_2_ZrCl_6_ powder. Since both Rb_2_ZrCl_6_:0.1%Te^4+^ and Rb_2_ZrCl_6_ powders appeared white under natural light, the nine digital patterns appeared white as a whole, representing the encryption process. When excited by 254 nm UV light, all nine numbers produced blue emission. Under excitation at 365 nm UV light, only the “520” filled with Rb_2_ZrCl_6_:0.1%Te^4+^ powder emitted yellow light, while other digits filled with Rb_2_ZrCl_6_ powder did not emit any light, this constituted the decryption process. The additional excitation and emission of luminescent information enhanced the overall security level, providing a more secure approach for anti-counterfeiting and confidential information encryption and decryption. Through this enhanced luminescent information excitation and emission process, the application prospects of Rb_2_ZrCl_6_:0.1%Te^4+^ in fields such as anti-counterfeiting and confidential information encryption and decryption were broadened.

We tested the stability of Rb_2_ZrCl_6_:*x*Te^4+^. The PL spectra of Rb_2_ZrCl_6_ powder, following a four-month exposure to atmospheric conditions (at 293 K and humidity levels of 20–30%), exhibited a 7% reduction in intensity when compared to freshly prepared powder. In contrast, the PL strength of Rb_2_ZrCl_6_:2%Te^4+^ decreased by only 6% after being stored for the same duration under identical environmental conditions (Figure S6). The exceptional stability of both Rb_2_ZrCl_6_ and Rb_2_ZrCl_6_:2%Te^4+^ could be attributed to their structural characteristics. Rb_2_ZrCl_6_ was a vacancy-ordered double perovskite, resulting from the combination of quaternary elements and vacancies; this unique structure contributes to its robust stability. In addition, Rb_2_ZrCl_6_ exhibited robust thermal stability both before and after the introduction of Te^4+^, as evidenced in Figure S7. Neither Rb_2_ZrCl_6_ nor Rb_2_ZrCl_6_:2%Te^4+^ underwent a phase transition, maintaining their structural integrity at temperatures up to 884 K, both before and after the doping process.

**Figure 7:**
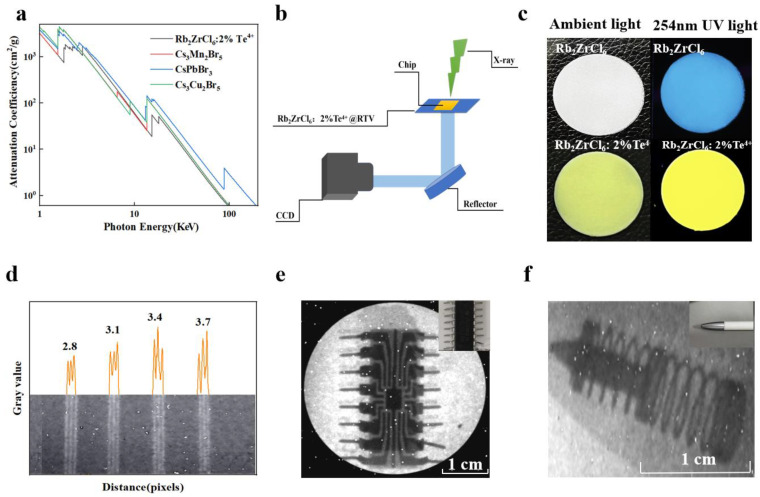
(**a**) X-ray absorption coefficients of Rb_2_ZrCl_6_:2%Te^4+^, Cs_3_Mn_2_Br_5_, CsPbBr_3_, and Cs_3_Cu_2_I_5_. (**b**) X-ray imaging prototype equipment system diagram. (**c**) Photographs of Rb_2_ZrCl_6_ and Rb_2_ZrCl_6_:2%Te^4+^ @RTV film under ambient light (**left**) and 254 nm UV light (**right**). (**d**) The graph of the gray value change in standard X-ray resolution test pattern plate. (**e**) X-ray image of the chip by using Rb_2_ZrCl_6_:2%Te^4+^ @RTV film as the scintillator screen. (The upper right corner of the picture is the chip picture). (**f**) X-ray image of the ballpoint pen by using Rb_2_ZrCl_6_:2%Te^4+^@RTV film as the scintillator screen. (The upper right corner of the picture is the ballpoint pen).

Additionally, a circular Rb_2_ZrCl_6_:2%Te^4+^@RTV thin film scintillation screen with a thickness of 70 μm and a diameter of 3 cm was also prepared, achieving a X-ray imaging spatial resolution of 3.7 lp/mm and a light output of 18,000 Photons/MeV for Rb_2_ZrCl_6_:2%Te^4+^, equivalent to 2.3 times that of BGO, demonstrating potential applications in X-ray imaging. Figure 7 showed the results of X-ray imaging testing.

Comparing Rb_2_ZrCl_6_, Rb_2_ZrCl_6_:2%Te^4+^, and commercial BGO in terms of XEL, it is observed that Rb_2_ZrCl_6_ achieves an optical yield of 89,000 photons/MeV, while Rb_2_ZrCl_6_:2%Te^4+^ reaches 18,000 photons/MeV, which is 11 times and 2.3 times of that of BGO, respectively, as shown in Figure S8. The future applications of Rb_2_ZrCl_6_:2%Te^4+^ and Rb_2_ZrCl_6_ in X-ray imaging were investigated. Considering that photodiodes generally peak in their responsiveness within the wavelength range of 500–600 nm [43]. Therefore, the emission light of Rb_2_ZrCl_6_ doped with Te^4+^ can better match the current mainstream CCD. Figure 7a compares the X-ray absorption capacity of Rb_2_ZrCl_6_:2%Te^4+^, Cs_3_Mn_2_Br_5_, CsPbBr_3_, and Cs_3_Cu_2_I_5_, Rb_2_ZrCl_6_:2%Te^4+^ showed similar X-ray absorption capacity to CsPbBr_3_. Figure 7b illustrates the X-ray imaging equipment utilized in this study. During the experimental procedures, the settings were configured with a voltage of 100 kV, a current of 1000 μA, and a peak energy of 165 kVp. The Rb_2_ZrCl_6_@RTV and Rb_2_ZrCl_6_:2%Te^4+^@RTV composite films with a diameter of 3 cm were prepared using a screen printing method. When observed under natural light, the Rb_2_ZrCl_6_ and Rb_2_ZrCl_6_:2%Te^4+^@RTV films appear white and light yellow respectively, and when excited by 254 nm UV light, they show blue and yellow respectively, as shown in Figure 7c. The thickness of Rb_2_ZrCl_6_ and Rb_2_ZrCl_6_:2%Te^4+^@RTV films is 70 μm, as shown in Figure S9. As shown in Figure 7d, Rb_2_ZrCl_6_:2%Te^4+^@RTV film as the scintillator screen is used to image the resolution plate, and a resolution of 3.7 lp/mm can be obtained. Its corresponding gray value is shown in Figure 7d. The resolution of Rb_2_ZrCl_6_@RTV film is 3.1 lp/mm, as shown in Figure S10. Figure 7e,f show the X-ray image of the chip and the ballpoint pen by using Rb_2_ZrCl_6_:2%Te^4+^@RTV film as the scintillator screen, showing clear internal structure. In the same way, the X-ray imaging test of Rb_2_ZrCl_6_@RTV film can also be used for the chip and the ballpoint pen imaging applications, as shown in Figure S11.

## 4. Conclusions

In summary, vacancy-ordered double perovskite Rb_2_ZrCl_6_ and Rb_2_ZrCl_6_:*x*Te^4+^ microcrystals were successfully prepared via hydrothermal method. The spectral properties of Rb_2_ZrCl_6_:*x*Te^4+^ were studied, and the application prospects of Rb_2_ZrCl_6_:*x*Te^4+^ in the fields of information encryption and X-ray detection are investigated. Rb_2_ZrCl_6_ has broadband STE emission, exhibiting a blue-white emission spectrum with fluorescence decay time and Stoker shift of 14.47 μs and 200 nm, respectively, without self-absorption. After doping, Rb_2_ZrCl_6_:*x*Te^4+^ presents different luminescence emissions at different excitation wavelengths. With the increase of Te^4+^ concentration, ^3^P_1_ to ^1^S_0_ emission of Rb_2_ZrCl_6_:*x*Te^4+^ gradually dominates, and the emission wavelength can be adjusted in the range of 455 nm to 575 nm, showing a transition from blue to yellow light, which means excellent spectral tunability. Rb_2_ZrCl_6_:*x*Te^4+^ microcrystals have broad spectrum emission, longer PL lifetime, and a significant Stokes shift. The strongest doping concentration, Rb_2_ZrCl_6_:2%Te^4+^, exhibits a strong yellow emission, and the decay time and Stokes shift of Rb_2_ZrCl_6_:2%Te^4+^ are 3.51 μs and 187 nm, respectively, attributed to its triplet self-trapping exciton emission. Therefore, Rb_2_ZrCl_6_:2%Te^4+^ can be applied to anti-counterfeiting and information encryption design, and can achieve specific color anti-counterfeiting and information encryption design by adjusting Te^4+^ concentration. For example, Rb_2_ZrCl_6_:0.1%Te^4+^ can produce different colors under different ultraviolet excitation energies, showing blue under 254 nm excitation and yellow under 365 nm excitation, which can realize the information encryption of blue and yellow color transformation. The application prospect in X-ray imaging was also investigated. The light yield of Rb_2_ZrCl_6_ and Rb_2_ZrCl_6_:2%Te^4+^ was 89,000 photon/MeV and 18,000 photon/MeV, respectively, which were 11 and 2.3 times that of BGO. Scintillation films with uniform thickness (70 μm) were prepared by combining the material with RTV, and spatial resolutions of 3.1 lp/mm and 3.7 lp/mm were achieved, respectively. Rb_2_ZrCl_6_:*x*Te^4+^ shows excellent application prospects in the field of information encryption and imaging.

## Data Availability

Data are contained within the article and Supplementary Materials.

## Supplementary Materials

The following supporting information can be downloaded at: https://www.mdpi.com/article/10.3390/ma17112530/s1, Figure S1: Absorption spectra of Rb_2_ZrCl_6_:*x*Te^4+^; Figure S2: PL spectrum of Rb_2_ZrCl_6:_ *x*Te^4+^ at 255 nm excitation; Figure S3: Temperature-dependent PL spectra of Rb_2_ZrCl_6_:1%Te^4+^ (λ_ex_ = 388 nm); Figure S4: Pseudo color map of Rb_2_ZrCl_6_:1%Te^4+^ PL spectra at low temperature (T= 80 –300 K, λ_ex_ = 255 nm); Figure S5: PLQY of sample Rb2ZrCl6 (λex = 255 nm) and sample Rb_2_ZrCl_6_:2%Te^4+^ (λex = 388 nm), respectively; Figure S6: Rb_2_ZrCl_6_ and Rb_2_ZrCl_6_:2%Te^4+^: Comparison of PL spectra of the fresh and 4 months after exposure to air. (temperature:293 K, humidity:20 ~ 30%). The insets show images of the corresponding fresh samples under 254 nm UV light; Figure S7: DSC and TGA curves of Rb2ZrCl6 and Rb_2_ZrCl_6_:2%Te^4+^, respectively; Figure S8: XEL curves for Rb_2_ZrCl_6_, Rb_2_ZrCl_6_:2%Te^4+^ and BGO; Figure S9: (a,b) respectively represent the thickness of the glass sheet, and the total thickness of the glass sheet and Rb_2_ZrCl_6_@RTV film. (c,d) respectively represent the thickness of the glass sheet, the total thickness of the glass sheet and Rb_2_ZrCl_6_:2%Te^4+^@RTV film; Figure S10: X-ray image of the resolution plate by using Rb_2_ZrCl_6_@RTV film as the scintillator screen; Figure S11: X-ray image of the chip (left) and the ballpoint pen (right) by using Rb_2_ZrCl_6_@RTV film as the scintillator screen.
