# Supplementary material for: Spectrally Tunable Lead-Free Perovskite Rb2ZrCl6:Te for Information Encryption and X-ray Imaging
Source: Materials (Basel). 2024 May 24;17(11):2530. doi: 10.3390/ma17112530 (PMC11173108; doi:10.3390/ma17112530)
Supplement: Supplementary file 1 [file materials-17-02530-s001.zip › materials-3024993-supplementary.pdf]

Supporting Information

Spectrally Tunable Lead-Free Perovskite Rb_2ZrCl_6:Te for Information Encryption and X-ray Imaging

Guoxue Pan, Mingqing Li, Xiaotong Yu, Yuanhao Zhou, Minghui Xu, Xinxin Yang, Zhan Xu, Qianli Li*, He Feng*

School of Materials Science and Engineering, Shanghai University, Shanghai 200444, China

*Corresponding author.

*Qianli Li: liqianli@shu.edu.cn

*He Feng: fh117@shu.edu.cn

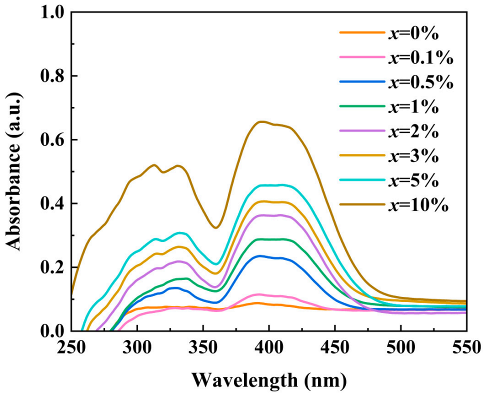

Figure S1. Absorption spectra of Rb_2ZrCl_6:$x$$Te^{4+}$.

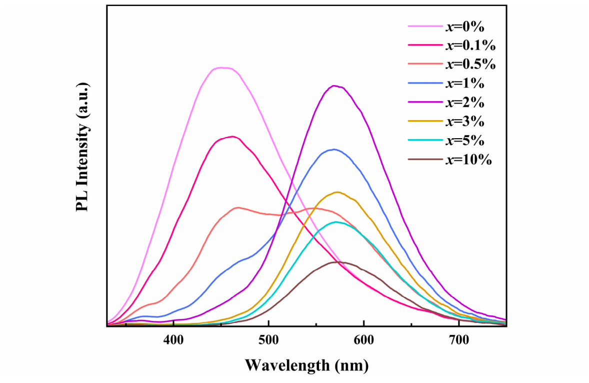

Figure S2. PL spectrum of Rb_2ZrCl_6: $x$$Te^{4+}$ at 255 nm excitation.

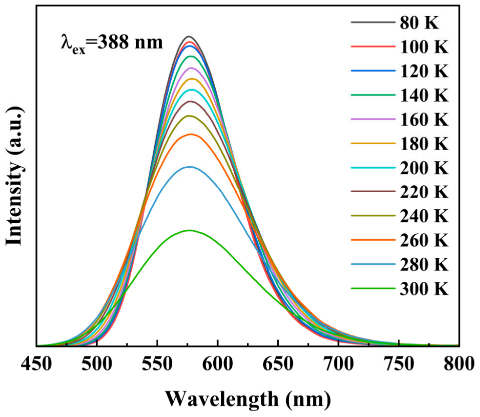

Figure S3. Temperature-dependent PL spectra of Rb_2ZrCl_6:1%Te^{4+} (λ_{ex} = 388 nm).

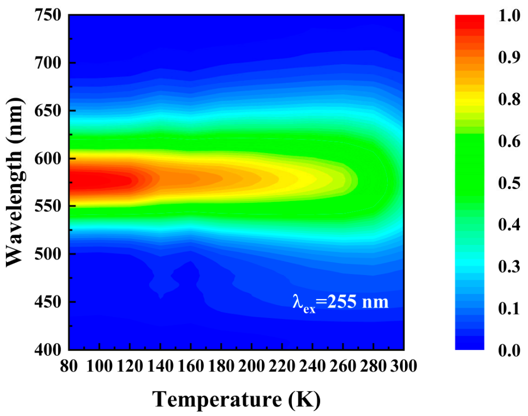

Figure S4. Pseudo color map of Rb_2ZrCl_6:1%Te^{4+} PL spectra at low temperature (T= 80 –300 K, λ_{ex} = 255 nm).

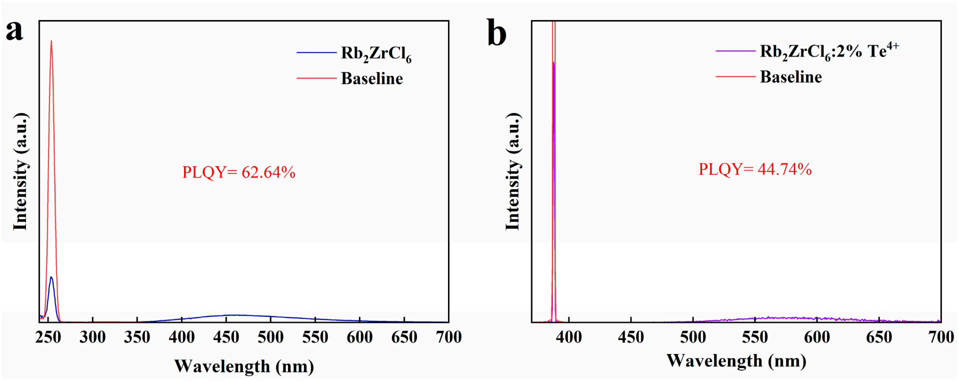

Figure S5. PLQY of sample Rb_2ZrCl_6 (λ_{ex} = 255 nm) and sample Rb_2ZrCl_6:2%Te^{4+} (λ_{ex} = 388 nm), respectively.

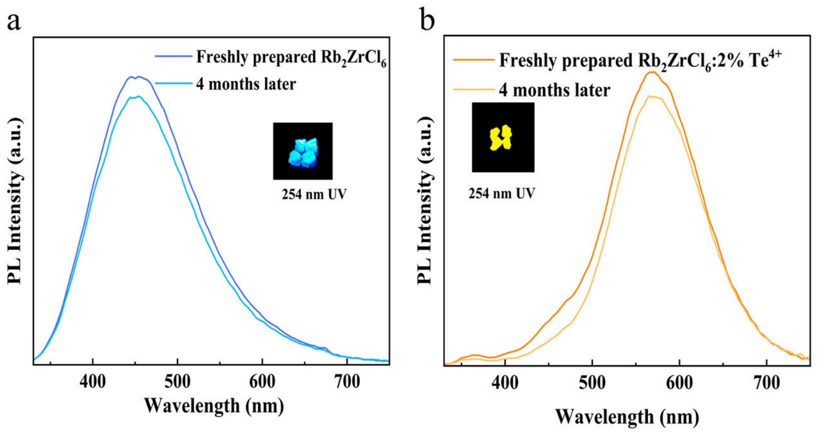

Figure S6. Rb_2ZrCl_6 and Rb_2ZrCl_6:2%Te^{4+}: Comparison of PL spectra of the fresh and 4 months after exposure to air. (temperature:293 K, humidity:20 ~ 30%). The insets show images of the corresponding fresh samples under 254 nm UV light.

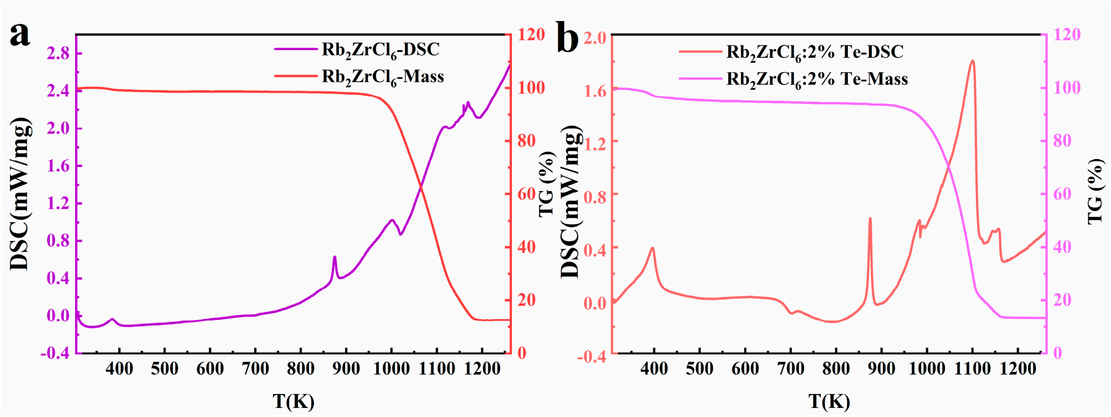

Figure S7. DSC and TGA curves of Rb_2ZrCl_6 and Rb_2ZrCl_6:2%Te^{4+}, respectively.

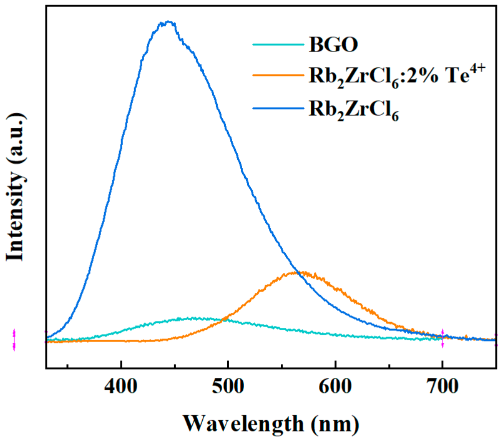

Figure S8. XEL curves for Rb_2ZrCl_6, Rb_2ZrCl_6:2%Te^{4+} and BGO.

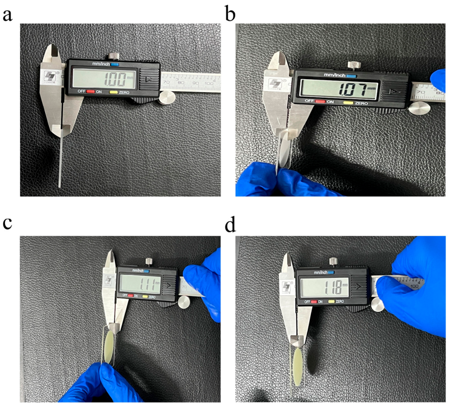

Figure S9. (a) and (b) respectively represent the thickness of the glass sheet, and the total thickness of the glass sheet and Rb_2ZrCl_6@RTV film. (c) and (d) respectively represent the thickness of the glass sheet, the total thickness of the glass sheet and Rb_2ZrCl_6:2%Te^{4+}@RTV film.

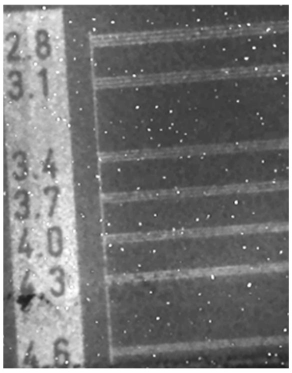

Figure S10. X-ray image of the resolution plate by using Rb_2ZrCl_6@RTV film as the scintillator screen.

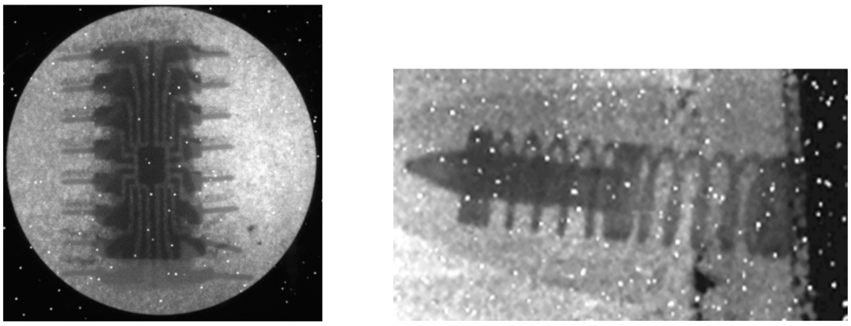

Figure S11. X-ray image of the chip (left) and the ballpoint pen (right) by using Rb_2ZrCl_6@RTV film as the scintillator screen.
